# An evaluation of pain experienced by patients during and after ultrasound-guided breast biopsy and patient coping strategies

**DOI:** 10.4314/gmj.v58i4.6

**Published:** 2024-12

**Authors:** Yaw B Mensah, Naa A Mensah, Hafisatu Gbadamosi, Linda Nketiah

**Affiliations:** 1 Department of Radiology, University of Ghana Medical School, Korle Bu, Accra, Ghana; 2 Department of Sociology, Leverhulme Centre for Demographic Science, University of Oxford, Oxford, UK; 3 Department of Radiology, Korle Bu Teaching Hospital, Korle Bu Accra, Ghana; 4 Imaging Department, University of Ghana Medical Centre, Legon, Ghana

**Keywords:** Core needle breast biopsy, pain, anxiety, ultrasound-guided, management

## Abstract

**Objective:**

To ascertain the experience and determinants of pain by breast biopsy patients and how the pain is managed in the first week following the procedure.

**Design:**

This was a panel longitudinal study design.

**Settings:**

The study was conducted at the Radiology Department of Korle Bu Teaching Hospital.

**Participants:**

The study participants comprised adult patients who presented to the Department of Radiology of Korle Bu Teaching Hospital for breast biopsy between 1 August 2022 and 31 January 2023.

**Main Outcome:**

The severity of biopsy-related pain, its associated factors and management were evaluated and documented.

**Results:**

The participants were between 21 and 81 years with a mean age of 48.1 years. There was no association between demographic and participant factors and the degree of pain experienced by the patient. There was a significant association between the radiologist's expertise (p<.001), blood pressure before the procedure (p=.026), quality of education given to the participant before the procedure (p<.001) and the degree of pain experienced.

**Conclusion:**

There was significant anxiety before the procedure. Most participants experienced mild pain, which did not interfere with daily activity. There was a significant association between participant pain and pre-procedure blood pressure, the radiologist's expertise and the quality of education participants received before the biopsy.

**Funding:**

None declared

## Introduction

Core needle biopsy (CNB) of the breast has gradually replaced excision biopsy and diagnostic needle localisation. This is because the procedure is accurate, relatively cheap, can be performed as an outpatient procedure and is associated with less morbidity, such as scarring.^1^,^2^ As a result, radiologists have an added role of overseeing patients' pre- and post-biopsy care.^3^

Like many invasive procedures, pain and anxiety are frequent challenges for many patients and, to some extent, the healthcare provider. This can significantly affect the patient's level of cooperation during the procedure. Patient anxiety is closely related to pain; hence, interventions that help to reduce anxiety during CNB can help reduce pain as well.^1^,^4^ Both physical and psychological factors influence the pain patients feel during biopsies. Factors like the patient's age, level of education, type of breast tissue, type, depth, and duration of the biopsy, amount of local anaesthetic, needle size, number of passes during the biopsy, and the expertise of the operator performing the procedure have all been evaluated in an attempt to find those that are significantly related to the pain. The most common factor that seems to influence the patient's experience is the expertise of the operator. 5–8 Studies have also substantiated the effectiveness of the use of local anaesthesia for pain control, reporting significantly lower pain levels with the use of local anaesthesia during breast biopsies.^9^

Currently, in most centres in Ghana, the radiology department manages the patient before, during, and immediately after the biopsy until the patient is ready to be discharged. The radiologist may or may not prescribe medication (for use while at home) for the patients, depending on their needs. Beyond giving patients pain relief and sometimes antibiotics, not much follow-up is done to find out about the patient's general well-being and pain management at home. To the best of the knowledge of the authors, no study in Ghana has documented follow-up findings in patients who have undergone breast biopsy. This study sought to ascertain the experience and determinants of pain by breast biopsy patients and how the pain (if any) was managed in the first week following the procedure.

## Methods

This study was a panel longitudinal design conducted between 1 August 2022 and 31 January 2023 at the Radiology Department of Korle Bu Teaching Hospital (KBTH), a 2000-bed hospital in Accra, Ghana. The department has a dedicated room for ultrasound-guided interventions, where biopsies of the breast and other organs are performed at least three days a week. The study subjects were adult patients who had been referred for an ultrasound-guided biopsy of one or both breasts on account of a suspicious lesion noted in a prior radiological imaging study.

Patients aged 18 years and above who had been referred for ultrasound-guided biopsy of the breast and were willing to be part of the study were recruited. Patients who had been referred for breast biopsy but did not want to take part in the study or were experiencing severe pain in the affected breast were excluded from the study

### Sample size determination

G*Power statistical software was used for the sample size calculations, and it employed the method of Hsieh and colleagues, ‘A simple method of sample size calculation for linear and logistic regression’.^10^ It was calculated based on the following parameter specifications:

(1) Level of significance, two-sided test at α = 0.05.
(2) Power (1-β) of 80%.
(3) 28% of patients develop pain and discomfort during breast biopsy, as documented by Seeley et al. in a related study published in 2016.
(4) Effect size: the minimum odds ratio (OR) considered to be clinically important (dependent on the factor of interest). The factor reported in the literature to be significantly associated with pain was “family history of breast cancer”. An OR of 2.00 was considered clinically important.
(5) a standard deviation of 0.5 for the exposure (given that the binary exposure follows a Bernoulli distribution with the probability of a subject achieving success, p, assumed to be equal to 0.5, the sample size was calculated from the formula: (p*(1-p))^0.5.

Based on these parameter specifications, the estimated sample size was 293. Allowing for a 10% attrition rate, 322 patients were to be recruited. A total of 334 participants were recruited for the study period.

### The technique of Ultrasound-guided biopsy of the breast

Four radiologists with 15 years, 8 years, 5 years and 1 year of experience performed the biopsies. Image guidance was achieved using a Toshiba Aplio 300 ultrasound unit with a high-frequency 7.5–12 MHz transducer. An initial ultrasound scan was performed to identify and characterise the lesion. Under the aseptic technique, the biopsy site was anaesthetised with 2-5mls of 2% lignocaine hydrochloride, depending on the depth of the lesion.

With the help of the ultrasound unit, the lesion was localised. The biopsy needle (G16) was then passed in the plane to the transducer into the lesion, and samples were taken. Between four and six samples were taken and immersed in a sample container with buffered formaldehyde. Haemostasis was achieved by applying pressure on the biopsy site. An adhesive tape was applied to the biopsy site over a sterile dressing. The participant was observed for 30 minutes and, when found fit, was given post-procedure instructions and then discharged.

### Data collection methods and instruments

Data collection for the study was done using a semistructured questionnaire which had three parts; A, information before biopsy; B, information during and immediately after biopsy; C, information after discharge on Days 1, 3 and 7 after the biopsy.

Part A was used to collect patient demographic information such as age, sex, educational level, marital status, occupation, history of breast pain, disease and/or biopsy. Part B was used to acquire information about the conduct of the technique and its effect on the participant. Information gathered included distance of lesion from skin, size of biopsy needle used, number of passes, volume and type of local anaesthetic agent used, degree of difficulty of biopsy, blood pressure and the patient's pain score using a 0-10 numeric pain intensity scale. Part C was used to document the degree of pain experienced by participants using a 0-10 numeric pain intensity scale, how the patient was managing the pain, the effect on quality of life/interference of daily activities and notable skin changes. This follow-up was intended for 30 days but was later changed to a seven-day follow-up because of significant attrition after the seventh day.

The data was analysed using the Statistical Package for the Social Sciences (SPSS) version 20. Participant demographic, clinical, and biopsy-related information was analysed using descriptive statistics like frequency and measures of central tendency, e.g., mean, and measure spread, e.g., standard deviation, to show the trend. Tests of associations, such as Pearson's chi-square tests, were used to determine if participants and other variables experienced associations between pain.

### Ethical Issues

Ethical approval was obtained from the Institutional Review Board of the Korle Bu Teaching Hospital (KBTH/MD/G3/22). All patients were given a detailed description, and both oral and written informed consent was obtained. Other ethical issues of confidentiality and anonymity were adhered to during the data collection and analysis. Patients were informed of their right of refusal to participate and assured that their refusal to participate would not affect any aspect or care given to them during the procedure.

**Definitions:**

1. Participants were then split into three groups: young adults (< 40 years), middle-aged (40-59) and elderly (60+).
2. The blood pressures of the participants were grouped as normal (less than or equal to 120/80mmHg), borderline (between 120-139/80-90 mmHg), high (140179/90-119mmHg) and very high (180/120mmHg or higher).
3. The pain scores obtained from the participants were regrouped into 0 as no pain, 1-3 as mild, 4-6 as moderate and 7-9 as severe and 10 as worst pain ever imaginable.^3^–^5^
4. The degree of interference with daily activity was based on the participant's own subjective interpretation of the extent to which his or her pain is preventing him or her from performing some routine activities he or she would have performed easily without the pain.
5. The degree of skin changes is the participant's subjective view of how severe the changes are compared to what he or she had before the procedure.
6. The participants' blood pressure (BP) was used as an index of patient anxiety, which is related in the literature.^11^ Because this study was primarily evaluating patient pain, we did not find it necessary to perform a detailed psychological test to check for anxiety so as not to prolong the interview for a patient who may be in pain after the procedure
7. The radiologist's expertise was indexed to the years the radiologist has been performing breast biopsies.

## Results

Three hundred thirty-four participants were recruited for the study, consisting of 332 females (99.4%) and 2 males (0.6%). The participants' ages ranged between 21 and 81, with a mean age of 48.1(11.8).

The characteristics of the participants are shown in Table 1. The middle-aged group was the most common age group, comprising 205 participants (61.5%). Most participants had some form of formal education, with the majority having tertiary education, 120 participants (37%) and 6 (1.9%) having no formal education. Most participants had no history of breast lumps; 214 (66%) and 110 participants (34.0%) had a history of breast lumps. In addition, 208 participants (64.2%) did not have breast pain, while the rest did. Thirty participants (9.3%) had a previous history of breast cancer, while 294 (90.7%) did not. There were 66 participants (20.4%) who had a previous history of breast biopsy, while the others did not.

**Table 1:** Characteristics of the participants

Variable	n (%)
**Age Group**	
**Young Adult (<40 yrs)**	71 (22.4)
**Middle aged (40-59 yrs)**	205 (61.5)
**Elderly (≥60 yrs)**	54 (16.1)
**Educational Attainment**	
**Primary**	36 (11.1)
**Secondary**	112 (34.6)
**Tertiary**	120 (37)
**Postgraduate**	50 (15.4)
**No Formal Education**	6 (1.9)
**Past History of Breast Lump**	
**Yes**	110 (34.0)
**No**	214 (66)
**Breast Pain**	
**Yes**	116 (35.4)
**No**	208 (64.2)
**Past History of Breast Cancer**	
**Yes**	30 (9.3)
**No**	294 (90.7)
**Past History of Breast Biopsy**	
**Yes**	66 (20.4)
**No**	258 (79.4)
**Quality of Education**	
**Poor**	0
**Satisfactory**	138 (41.5)
**Good**	166 (49.6)
**Very Good**	30 (8.9)
**Immediate Post Procedure Pain**	
**No Pain**	112 (34.6)
**Mild Pain**	116 (35.8)
**Moderate Pain**	78 (24.1)
**Severe Pain**	16 (4.9)
**Worst Pain Ever**	2 (0.6)

One hundred and sixteen participants (34.6%) complained of mild pain after the procedures, with two participants (0.6%) describing theirs as the worst pain ever imaginable, Table 1.

The mean and median pain scores were 2.2 ± 2.2 and 2, respectively. Following the biopsy, 138 participants (41.5%) thought the education they received before the procedure was satisfactory, while 166 participants (49.6%) thought it was good,

As much as possible, patients were made to relax and spoken to till the BP returned to normal before the procedure was done. The procedure was cancelled if the BP did not return to normal, and the participant was given a note to go back to their doctor for further management. The initial BP was normal in 132 participants (40.4%), borderline in 68 participants (21.2%), high in 118 participants (35.9%) and very high in 8 participants (2.1%), as shown in Table 2. The BP measured immediately after the procedure is also shown in Table 2. The participants had lower blood pressure after the procedure than before, and the difference was statistically significant (χ^2^ ([12], N = [334]) = [97.978^a^], p <[.001].

**Table 2:** Blood pressure of participants before and after biopsy

Blood Pressure	Before Procedure n (%)	After Procedure n (%)
**NORMAL**	66 (40.4)	84 (52)
**BORDERLINE**	34 (21.2)	37(23)
**HIGH**	58 (35.9)	41(25)
**VERY HIGH**	4 (2.1)	

n (%)- Number of Participants (Percentage)

There were slightly more lesions in the right breast, 158 (48.8%), than in the left 146(45.1%), with 9 (5.6%) participants having lesions in both breasts. Of the unilateral lesions, 135 participants (88.2%) had lesions in only one breast area, while 18 participants (11.0%) had multicentric/multifocal lesions. The radiologists performing the procedure reported that the biopsy was not difficult in 218 participants (67.3%), slightly difficult in 90 participants (27.8%), moderately difficult in 14 participants (4.3%) and very difficult in two participants (0.6%). The mean number of number needle passes/cores taken was 4.9, and the median was 5, with a maximum number of 9 passes, as shown in Table 3.

**Table 3:** Number of needle passes per biopsy

Number of Needle passes/Cores	Number Participants n (%)
**1**	5 (1.4)
**2**	7 (2.1)
**3**	26 (7.7)
**4**	89 (26.6)
**5**	105 (32.5)
**6**	72 (21.7)
**7**	19 (5.6)
**8**	9 (2.8)
**9**	2 (0.6)

Nearly all the patients had approximately 3ml of 2% plain xylocaine. The average depth of the lesions from the skin was 1.51 ± 1.24cm. Participants were followed up with phone calls to assess their well-being regarding the degree of pain they experienced, their remedy for the pain, the extent to which the pain interfered with their daily activities, and any skin changes resulting from the biopsy. A total of 224, 222, and 198 participants were reached on the first, third, and seventh day after the procedure, respectively. The information obtained has all been summarised in Table 4. Apart from pain and skin changes, participants reported itching, bloody nipple discharge, a bigger lump, and a tingling sensation. Ten participants (4.5%) reported these symptoms, putting them in the minority yet an important aspect to report.

**Table 4:** Patient evaluation first seven days after biopsy

Variable	POD 1 n (%)	POD 3 n (%)	POD 7 n (%)
**Total Number of Participants**	224	222	198
**Degree of Pain**			
**No Pain**	44 (24.1)	118 (53.2)	175 (88.3)
**Mild Pain**	114 (50.9)	80 (36.0)	21 (10.6)
**Moderate Pain**	44 (19.6)	24 (10.8)	2(1.0)
**Severe Pain**	8 (3.6)	0	0
**Worst Pain Ever**	4(1.8)	0	0
**Pain Management**			
**Pain Medication**	185 (84.4)	58 (17.9)	14 (14.1)
**Other (Warm Compress, Balm etc.)**	2(0.9)	1 (0.5)	1 (0.5)
**Skin Changes**			
**No**	148 (65.7)	168 (75.7)	178 (89.9)
**Mild**	70 (31.4)	42 (18.9)	20 (10.1)
**Moderate**	2 (1)	12 (5.4)	0
**Severe**	4(1.9)		0
**Interference with daily activities**			
**No**	190 (84.4)	190 (84.4)	194 (98)
**Mild**	24(11)	24 (11)	2(1.0)
**Moderate**	8 (3.7)	8 (3.7)	2(1.0)
**Severe**	2(0.9)	2 (0.9)	0

n (%)- Number of Participants (Percentage)

POD - Post Procedure Day

The Pearson Chi-square test was used to check for the association between other variables and the degree of pain experienced after the biopsy. The severity of the pain experienced reduced from the day of the procedure to day 7. Though this difference was obvious from the observed values, it was not statistically significant. The difference between interference to their daily activities a day after the procedure and that on the seventh day was statistically significant. These and the association between the rest of the variables and the degree of pain experienced have been documented in Table 5.

**Table 5:** Test of association between various variables and degree of pain experienced after the biopsy

Variables	Degree of Pain Experienced		
	Chi-square Value	df	Sig
**Number of Days post procedure**	36.688^a^	45	.807
**Interference with daily activities**	80.977^a^	12	<.001
**Marital Status**	37.682^a^	40	.575
**Educational Status**	45.622^a^	50	.650
**Occupation**	71.341^a^	90	.926
**Past History of Breast Cancer**	9.564^a^	20	.975
**Previous of Breast Biopsy**	.928^a^	20	.462
**Location of lesion within breast**	118.736^a^	150	.972
**Depth of Lesion in the Breast**	548.361^a^	530	.282
**Number of Needle passes**	95.241^a^	100	.616
**Degree of difficulty**	22.899^a^	40	.986
**BP - Before**	59.187^a^	40	.026
**BP -After**	24.208^a^	30	.763
**Pre-procedure education**	84.740^a^	30	<.001
**Radiologist Expertise**	134.005^a^	40	<.001

After the day 1 interview, participants were asked if they had any concerns they needed answers for. One hundred and fifty-seven participants (70%) wanted to know when they would receive their biopsy reports, where to go for the report and what to do with it. They also admitted to being anxious about the outcome of their biopsy. One hundred and sixty-eight participants (75%) out of the 224 participants interviewed on the first day after the procedure showed gratitude to the research assistant for calling to follow up on them.

## Discussion

Image-guided biopsy has become the method of choice for evaluating suspicious breast lesions in most centres, with ultrasound being the preferred modality for guidance. 3 A total of 334 participants were recruited for the study, including 332 females (99.4%) and 2 males (0.6%). The participants' ages ranged between 21 and 81, with a mean age of 48.1 + 11.8. A study in the USA found a similar mean age of 48.2 years in its investigation of factors likely to lead to distress associated with benign breast biopsy.^12^ A relatively similar study by Humphrey et al. documented an age range of 22 to 88 years. Their mean age, 51.4 years, was, however, slightly higher than what pertained in this study, which could be explained by the larger Caucasian composition of their population. Indeed, studies have suggested a slighter higher age of breast cancer in Caucasians than in their counterparts of African descent.^7^,^8^,^13^

The level of anxiety before the procedure was estimated with their pre- and post-procedure blood pressure levels. The study revealed that the participants had significantly lower blood pressure after the procedure than before, confirming anxiety before the procedure. It is worth noting that the patients with high blood pressure were calmed and counselled, which led to their blood pressure reducing to normal levels before the procedure was performed, making the BP a fair index of patient anxiety level. Several studies support the fact that there is significant anxiety associated with breast biopsy. This is experienced before, during and after the procedure and can adversely affect patients' perception of breast biopsy and short-term quality of life.^1^,^5^,^7^–^9^,^14^ Therefore, it is not surprising that some of the participants had high blood pressure before the procedure and that some 25% of the participants had high blood pressure after the procedure.^6^ and this could have been induced by the pain they experienced due to the procedure.

There was no significant association between the degree of pain experienced by the participants and patient factors such as age, employment status, occupation, marital status, past history of breast cancer, previous biopsy experience, location of the lesion within the breast and blood pressure after the procedure. Similarly, there was no significant association between the degree of pain experienced by the participants and technique-related factors such as degree of difficulty and depth of the lesion within the breast. Several studies have documented that, just like this study, demographic and patient factors are not significantly associated with the degree of pain experienced by the patient.^3^,^5^,^7^,^13^ Seely et al. and Humphrey et al. have, however, documented that younger patients and less educated patients tend to have more pain than their older counterparts, which was not supported by this study.^5^,^7^,^13^ The difference could be due to the different cultural settings in which these studies were undertaken and the demographic characteristics of the study population.

However, there was a significant association between the degree of pain experienced by the participant and the expertise of the radiologist performing the procedure, the blood pressure before the procedure (an index of the level of anxiety) and the quality of education the patient gave. Like this study similar studies have reported that there is a significant association between the expertise of the one performing the biopsy and the degree of pain experienced by the patient.^3^,^15^ However, other studies have failed to confirm this finding because they believe that when well trained, there will not be any difference in how radiologists perform biopsies and their effect on patient experience.^3^,^5^,^16^

The study did not evaluate the relationship between the difficulty of the procedure and the expertise of the radiologist as it would have required the team to consider and eliminate factors such as the patient's breast size, anxiety level, and previous experience, among others, which could be important confounding factors. The quality of education and communication with the patient is vital with respect to the pain and anxiety experienced by the patient undergoing a biopsy, and several other studies have also confirmed this.^4^,^9^,^12^,^14^,^16^,^17^

In this study, the median and mean participant pain scores were 2 and 2.2, respectively. This was in the mild range and was consistent with what many similar studies have documented.^1^,^5^–^7^,^12^,^14^ Therefore, it is reasonable to expect that the biopsy only interfered with the activity of about 16% of the participants during the first three days after the procedure and reduced to 2% by day 7. This may also be supported by the fact that while slightly more than half of the participants took medication to manage their pain/discomfort in the first three days, only 14% continued to use pain remedies by day 7.

Apart from the pain the participants experienced, there were skin changes worthy of note in about 34% of the participants on day 1, which reduced to 16% at the end of the first week. In addition, a small number of participants (4.5%) had other symptoms such as itching, tingling sensation of the breast, bloody nipple discharge etc. Though these participants were in the minority, their complaints could not be overlooked as these could affect their outlook on the procedure should it become necessary. Their perception could also influence that of peers in the long term. It is worth noting that 75% of the participants called were appreciative of the follow-up. Asked if they had any questions, 70% of the participants wanted to know when they would receive their biopsy reports, where to go for the report and what to do with the report. These underscore the fact that patients need more care than just managing their pain and infection prevention.

Studies have confirmed the fact that patient anxiety can really affect patients' quality of life for various reasons, prominent among them being positive histology reports.^5^,^9^,^12^,^14^,^16^ This means that patients who undergo biopsies and other similar minimally invasive procedures in the hospital should be provided a comprehensive multidisciplinary package of care, which should include psychological support for unexpected biopsy outcomes.

This would help manage both the physical and psychological challenges of such patients.

A major limitation of this study was that nearly a third of the study population could not be reached for the postprocedure interview. This could be due to the fact that some contact numbers the research team had was that of the one who sought the appointment for the patient and not that of the patient. Though a few of these individuals opted to answer the questions, the research team could not independently verify their responses and, therefore, did not include them in this study. The team thought there was a strong possibility that their responses would be based on their assessment of the participant's condition viz a viz the participant's own feelings. The absence of a control group in this study was a limitation which could have made the inferences a little more objective. However, the team is confident that the questionnaire used in the assessment made the study very objective.

There was a significant association between the degree of pain experienced by the participants and patient anxiety before the procedure, the education they received before the procedure, and the expertise of the radiologist. Participants were appreciative of the follow-up calls, which allowed them to ask questions such as where and when they will obtain their histopathology results and what to do with them.

Percutaneous Trucut breast biopsies have come to stay as a means of diagnosing breast cancer. Steps should be taken to reduce patient anxiety (before and after the procedure) and pain during the procedure, which should start from the referring physician who initiates the biopsy process. Institutions that undertake breast biopsies should have a comprehensive multidisciplinary care package for patients before, during and after the biopsy to ensure optimum patient experience and care.

## Conclusion

There was significant anxiety before the procedure, which was reduced after adequate patient education and communication. Most participants experienced mild pain, which did not significantly interfere with their daily activities.

## References

[1] Soo MS, Jarosz JA, Wren AA (2016). Imaging-Guided Core-Needle Breast Biopsy: Impact of Meditation and Music Interventions on Patient Anxiety, Pain, and Fatigue. Journal of the American College of Radiology.

[2] CardoȘ RAI, Șoflău R, Gherman A, Sucală M, Chiorean A (2017). A mobile intervention for core needle biopsy related pain and anxiety: A usability study. J Evid Based Psychother.

[3] Sharma S, White C, Appavoo S, Yong-Hing CJ (2024). Optimizing Patient-Centered Care in Breast Imaging: Strategies for Improving Patient Experience. Acad Radiol.

[4] Vasan A, Baker JA, Shelby RA, Soo MSC (2017). Impact of Sodium Bicarbonate-Buffered Lidocaine on Patient Pain During Image-Guided Breast Biopsy. Journal of the American College of Radiology.

[5] Seely JM, Hill F, Peddle S, Lau J (2017). An evaluation of patient experience during percutaneous breast biopsy. Eur Radiol.

[6] Prabhu VG, Sprouse HA, Brignull CG (2024). The Impact of Virtual Reality on Anxiety and Pain During US-Guided Breast Biopsies: A Randomized Controlled Clinical Trial. J Breast Imaging.

[7] Pang E, Crystal P, Kulkarni S, Murphy K, Menezes RJ (2016). An Audit of Pain Experienced During Image-Guided Breast Biopsy Procedures at an Academic Center. Canadian Association of Radiologists Journal.

[8] Soliman AH (2022). Ultrasound-guided vacuum-assisted excision biopsy in breast fibroadenomas: an Egyptian center experience. Egyptian Journal of Radiology and Nuclear Medicine.

[9] Soo AE, Shelby RA, Miller LS (2014). Predictors of pain experienced by women during percutaneous imaging-guided breast biopsies. J Am Coll Radiol.

[10] Hsieh FY, Bloch DA, Larsen MD (1998). A simple method of sample size calculation for linear and logistic regression. Stat Med.

[11] Al-Shimmery Haydar A, Raheem Alyaa (2022). Influence of Anxiety and Claustrophobia on Blood Pressure and Heart Rate during MRI Scan. Medico Legal Update.

[12] Kotwal AA, Schonberg MA (2017). Cancer Screening in the Elderly: A Review of Breast, Colorectal, Lung, and Prostate Cancer Screening. Cancer J.

[13] Humphrey KL, Lee JM, Donelan K (2014). Percutaneous Breast Biopsy: Effect on Short-term Quality of Life. Radiology.

[14] Soo MS, Shelby RA, Johnson KS (2019). Optimizing the Patient Experience during Breast Biopsy. J Breast Imaging.

[15] Ashour ASA, Abd-ElGawad M, Yohanna M (2022). Is music intervention effective in reducing anxiety and pain during breast biopsy procedure? A systematic review and meta-analysis of randomized controlled trials. Support Care Cancer.

[16] Miller LS, Shelby RA, Balmadrid MH (2013). Patient anxiety before and immediately after imaging-guided breast biopsy procedures: Impact of radiologist-patient communication. Journal of the American College of Radiology.

[17] Carney PA, Kettler M, Cook AJ (2009). An Assessment of the Likelihood, Frequency, and Content of Verbal Communication Between Radiologists and Women Receiving Screening and Diagnostic Mammography. Acad Radiol.

